# Relationship between serum inflammatory cytokines and suicide risk in patients with major depressive disorder

**DOI:** 10.3389/fpsyt.2024.1422511

**Published:** 2024-06-27

**Authors:** Yiyue Yang, Kaiqi Gu, Jing Li

**Affiliations:** ^1^ Department of Psychiatry, the First Affiliated Hospital of Chongqing Medical University, Chongqing, China; ^2^ Sichuan Provincial Center for Mental Health, Sichuan Provincial People’s Hospital, School of Medicine, University of Electronic Science and Technology of China, Chengdu, China; ^3^ Sleeping and Psychosomatic Center, Dazu District People’s Hospital, Chongqing, China

**Keywords:** depression, inflammation, cytokines, suicide risk, MDD (major depressive disorder)

## Abstract

**Background:**

Studies have shown that increased inflammatory cytokines are associated with suicide risk, but the relationship between suicide risk and inflammatory cytokines is not clear. This study aimed to investigate the relationship between specific inflammatory markers and suicide risk in patients with MDD.

**Methods:**

This is a cross-sectional study. Firstly, we measured and compared psychological characteristics and 10 peripheral inflammatory cytokines in 130 MDD patients and 130 healthy controls(HC). Secondly, MDD patients were divided into 4 groups according to the severity of suicide risk for comparison between groups. Finally, multiple linear regression analysis was used to explore the predictors of suicide risk.

**Results:**

We found that the group with higher suicide risk had higher levels of IL-6, CRP, TNF-α, CXCL-2, and IFN-γ, and lower levels of IL-2 and IL-8 (all p<0.01). However, we found no difference in CRP between MIS and LS groups (p=0.337). Regression models were well-fitted. IL-2,IL-8 negatively predicted suicide risk (all p<0.05),IL-6,CRP,TNF-α,CXCL-2, and IFN-γ can positively predict the risk of suicide (all p<0.05).

**Limitations:**

This study employed a self-assessment scale.

**Conclusions:**

The higher the levels of IL-6, CRP, TNF-α, CXCL-2, and IFN-γ and the lower the levels of IL-2 and IL-8 of MDD patients, the higher the risk of suicide.

## 1 Introduction

Suicide is a major public health problem worldwide. It is the leading cause of death in patients with depression worldwide, with 800,000 patients dying by suicide each year, according to the World Health Organization (1). Major depressive disorder (MDD) is a chronic mental illness that leads to social and occupational disabilities (2, 3) and is highly associated with suicide (4). Studies have shown that severe depression increases the risk of suicide by 20 times (5). Therefore, it is particularly important to adopt some suicide prevention strategies to effectively avoid suicide deaths (6).

Previous studies have shown that inflammatory processes are linked to the pathophysiology of MDD (7). Inflammatory cytokines contribute significantly to the neurologic progression of MDD (8). Studies have shown that the peripheral blood of first-episode MDD patients was in an inflammatory state, such as the increase of interleukin-1β (IL-1β), interleukin-6 (IL-6), tumor necrosis factor-α (TNF-α) and interferon-γ (IFN-γ) and the decrease of interleukin-2 (IL-2) (9).

Although the neurobiological factors behind suicide are unclear, recent studies suggest that inflammatory cytokines may play a role in the pathophysiology of suicide (5, 10, 11). A 15-year follow-up study of Taiwanese adults identified cytokines of peripheral inflammation as potential biomarkers of suicide risk (12). So far, the most significant changes in inflammatory cytokines found by studies pointing to suicide are: (1) increases in IL-6, IL-1β, IL-4, IL-10, IL-13, and tumor necrosis factor-α (TNF-α) (8, 13, 14). Among them, IL-6 is most closely associated with suicide (15). (2) IL-2 (5)and IL-8 (11) are decreased. (3) C-reactive protein (CRP) increased. It has been suggested that hs-CRP is a potential inflammatory cytokine for suicide attempts in patients with depression (16). A 2019 study concluded that anti-inflammatory drugs reduce the risk of SI, providing the first proof of concept of suicide prevention targeting inflammatory pathways (17). A study that summarized nearly 5 years of suicide-related biomarkers found that the effectiveness of the biomarkers in diagnosing and predicting suicide was significant. Low levels of tryptophan separate suicidal ideation from other mental health problems, elevated levels of cortisol separate suicidal ideation from suicide attempts, and endocannabinoid levels separate past suicide attempts from current suicidal ideation and other mental health problems. The increase of CRP level can predict the increase of the severity of suicidal ideation and suicide attempt, but there are still differences between individuals (18).

At present, there is increasing evidence of an association between inflammatory cytokines and suicide (5), but the data provided in the literature remains inadequate and inconsistent. Although many studies support these inflammatory factor changes, there are still studies that deny the association between suicide and them (14). There is also a lack of studies on the relationship between inflammatory cytokines and suicide risk in MDD patients. Therefore, such studies need to be expanded to provide more precise new ideas on clinical intervention and prevention strategies for MDD subtypes associated with suicidal ideation. In addition, previous studies focused on the relationship between inflammatory markers in MDD patients with or without suicidal ideation, and there were few studies on inflammatory cytokines in MDD patients with different suicide risk groups.

Therefore, this study aims to study the changes of inflammatory cytokines in the peripheral blood of MDD patients with different degrees of suicide risk, to explore the relationship between inflammatory cytokines and suicide risk in MDD patients, which helps provide theoretical support for the precise intervention of MDD patients with different degrees of suicide ideation. The main research questions are: (1) What inflammatory cytokines are associated with MDD? (2) What inflammatory cytokines are associated with suicide? (3) What is the relationship between the severity of suicide risk and specific inflammatory cytokines in patients with MDD?

Based on the results of previous studies (9, 19) We made our hypothesis: (1) Both MDD and suicide are associated with IL-1β, IL-2, IL-6, IL-8, IL-10, IFN-γ, TNF-α, CRP, CXCL-1 and CCL2. (2) The higher the risk of suicide, the higher the levels of IL-1β, IL-6, IL-10, IFN-γ, TNF-α, CRP, CXCL-1, and CCL2, and the lower the levels of IL-2 and IL-8.

## 2 Methods

### 2.1 Participants and data collection

The study was approved by the ethics committee of the First Affiliated Hospital of Chongqing Medical University. We recruited 130 MDD patients(Diagnosed by two psychiatrists with attending physician titles or above) from the psychiatric outpatient Department of the First Affiliated Hospital of Chongqing Medical University from March 2022 to March 2023. According to the paired case-control method, we recruited 130 healthy control(HC) subjects in a 1:1 ratio. All participants provided written informed consent. Before the survey, all the researchers were trained on the survey steps, methods and measurement tools. After the survey, the consistency test was conducted, and the r>80%. Psychiatrists structured interviews for all participants to rule out the extra mental illness, by a medical center for all participants to body check rule out other conditions. Inclusion criteria of the test group: (1) Meet the diagnostic criteria for unipolar depressive disorder (ICD-11) and Beck Depression Inventory (BDI) ≥8. (2) Never take antidepressants. (3) 18–60 years old with junior high school or above education level. (4) Able to cooperate with the physical examination before enrollment, understand and cooperate with the questionnaire filling, and venous blood extraction. Exclusion criteria of test group: (1) suffering from a serious physical illness. (2) suffering from other mental illnesses. (3) Major surgery, chronic inflammation, or persistent fever within 3 months. (4) Taking hormonal medications. (5) Pregnant or lactating women. (6) acute infection status. Inclusion criteria for the healthy control group: (1) be in good health and have never suffered from mental illness. (2) 18–60 years old with junior high school or above education level. (3) Able to cooperate with the physical examination before enrollment, understand and cooperate with filling out the questionnaire, and draw venous blood. Exclusion criteria for the healthy control group: (1) Major surgery, chronic inflammation, or persistent fever within 3 months. (2) Taking hormonal medications. (3) Pregnant or lactating women. (4) patients with substance dependence or drug use history. (5) acute infection status. Participants were given a psychological questionnaire and their serum levels of inflammatory cytokines were measured using enzyme-linked immunosorbent assay (ELISA). Psychological tests and blood collection were performed at the same time point.

### 2.2 Measures

#### 2.2.1 Demographic data

We used a self-designed scale to collect demographic data of participants, including name, age, sex, telephone number, years of education, etc.

#### 2.2.2 Suicide risk

We used the Nurses’ Global Assessment of Suicide Risk Scale (NGASR), a reliable tool for assessing suicide risk (20–22), to assess participants’ suicide risk. At present, the Chinese version of NGASR is widely used in psychiatric research (23, 24). It has 15 entries, each with a “yes” or “no” option. “Yes” is worth 1 or 3 points, and “none” is worth 0 points. Finally, the total score is calculated according to the extra points rule. A higher score indicates a higher risk of suicide. In this study, MDD patients were grouped according to the NGASR score. Low suicide risk (LS) group ≤5 points, Mild suicide risk (MIS) group 6–8 points, Moderate suicide risk (MOS) group 9–11 points, Severe suicide risk (SS) group ≥12 points (25).

#### 2.2.3 Depressive symptoms

We chose the Beck Depression Inventory (BDI-13) to assess the level of depressive symptoms. This scale has good reliability and validity (26, 27), which contains 13 items. The subjects were given answers based on the presence and severity of symptoms (0–3 scale). The total score <4 is classified as no depressive symptoms, 5–7 as mild depressive symptoms, 8–15 as moderate depressive symptoms, and >15 as severe depressive symptoms. MDD patients in this study required a BDI score ≥8.

#### 2.2.4 Determination of serum inflammatory cytokines

5 ml of venous blood samples were taken from participants and placed in a serum separation tube. The serum was separated by centrifugation for 10 minutes at 3500r/min within 2 hours. The supernatant was extracted into a cryopreserved tube and stored in a freezer at -20°C. We repeatedly determined human IL-1β, IL-2, IL-6, IL-8, IL-10, IFN-γ, TNF-α, CRP, CXCL-1, and CCL2 levels by enzyme-linked immunosorbent assay (ELISA) using a commercial Jiubang bio-kit(Biosine Biological Products Company, Chongqing, China.).

### 2.3 Statistical analyses

SPSS software (version 26) was used for statistical analysis. First, to study depression-related inflammatory cytokines, we divided the participants into the MDD group and the healthy controls(HC) group and conducted descriptive statistics. The measurement data met the normal distribution and an independent sample T-test was adopted, and the results were expressed as M ± SD. The count data were compared between groups by χ2 test, and the results were expressed as n (%). Inflammatory cytokines with no difference between the two groups will be excluded before the second step of data analysis. Second, to explore the inflammatory cytokines related to suicide, we divided the MDD group into four groups according to the level of suicide risk and used one-way ANOVA to compare the inflammatory cytokines of the four groups, followed by *post-hoc* pairwise comparisons for pairwise comparison. Third, Pearson correlation analysis was used to analyze the age, years of education, total score of suicide risk, and related inflammatory cytokines of the MDD patients group. As the correlation coefficient gets closer to 1, the variables become more correlated with each other (28). Fourth, we used multiple linear regression analysis to explore the predictors of suicide risk with suicide risk as the dependent variable, associated inflammatory cytokines as the independent variable, and age and years of education as the covariable. All the analysis with P < 0. 05 for the difference was statistically significant.

## 3 Results

### 3.1 Comparison of demographicl data between the HC group and MDD group

The distribution characteristics of participants are shown in (**Table 1**). In terms of general information, years of education, BDI, and NGASR in the MDD group were significantly higher than those in the HC group (all p<0.001). The MDD group was younger than the HC group (p<0.001). There was no significant difference in gender between the two groups (p=0.330).

**Table 1:** Comparison of general data and inflammatory cytokines levels test results between the two groups $\left(\bar{x}\pm s\right)$ .

	MDD group (n=130)	HC group (n=130)	Omnibus Test
General information	M±SD	M±SD	*p-*value
Age (years)	30.39**±**9.56	38.11**±**8.56	<0.001
Gender (n,%)	/	/	0.330
Male	20 (15.4%)	26 (20%)	/
female	110 (84.6%)	104 (80%)	/
Years of Education	15.24**±**1.72	13.50**±**3.13	<0.001
BDI	16.52±5.39	1.76±1.65	<0.001
NGASR	7.97±3.44	1.36±1.13	<0.001

MDD, major depressive disorder; HC, healthy controls; M, mean; SD, standard deviation.

### 3.2 Comparison of inflammatory cytokines levels between the HC group and MDD group

**Figure 1:**
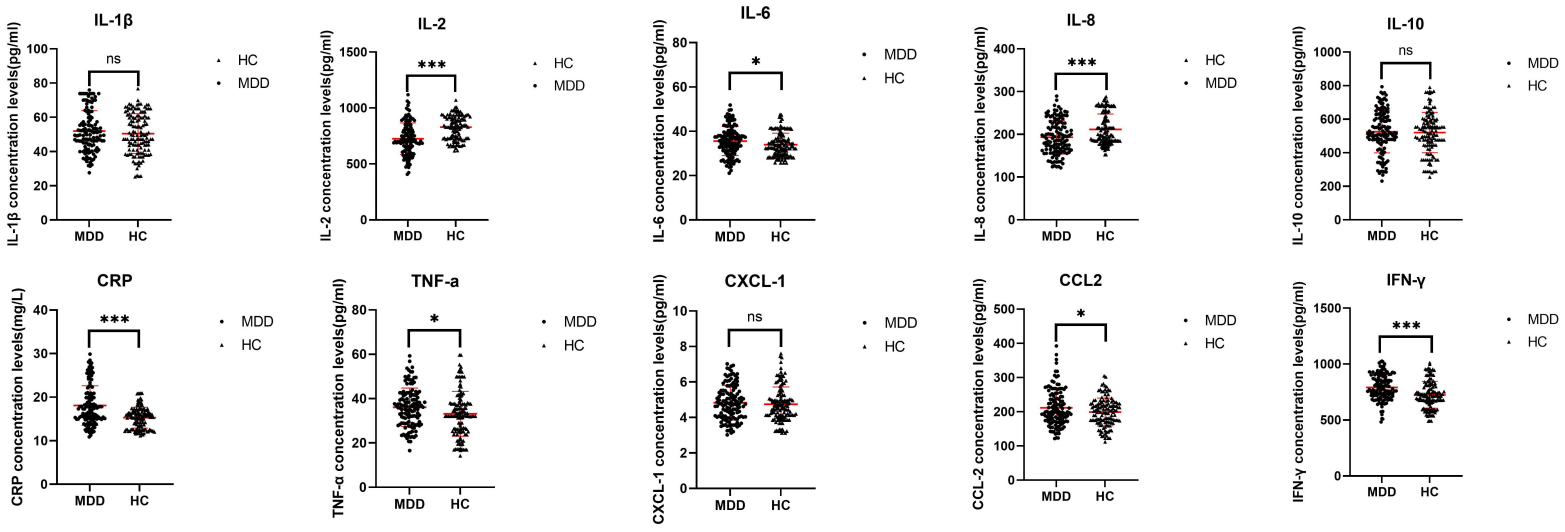
Comparison of serum inflammatory cytokines between MDD group and HC group. The red line represents the M±SD. MDD - major depressive disorder; HC - healthy controls. ns- not significant (P>0.05). *- significant correlation (P<0.05). **- significant correlation (P<0.01). ***- significant correlation (P<0.001).

In terms of levels of inflammatory cytokines, IL-6, CRP, TNF-α, CCL2, and IFN-γ were significantly higher in the MDD group than in the HC group (p=0.027,p<0.001,p=0.013,p=0.035,p<0.001). The levels of IL-2 and IL-8 in the MDD group were lower than those in the HC group (all p<0.001).Two groups of IL-1, IL-10 and CXCL-1 had no obvious difference (p=0.305, p=0.741, p= 0.507)(see **Figure 1**).

### 3.3 Comparison of levels of inflammatory cytokines in MDD grouped by suicide risk

**Figure 2:**
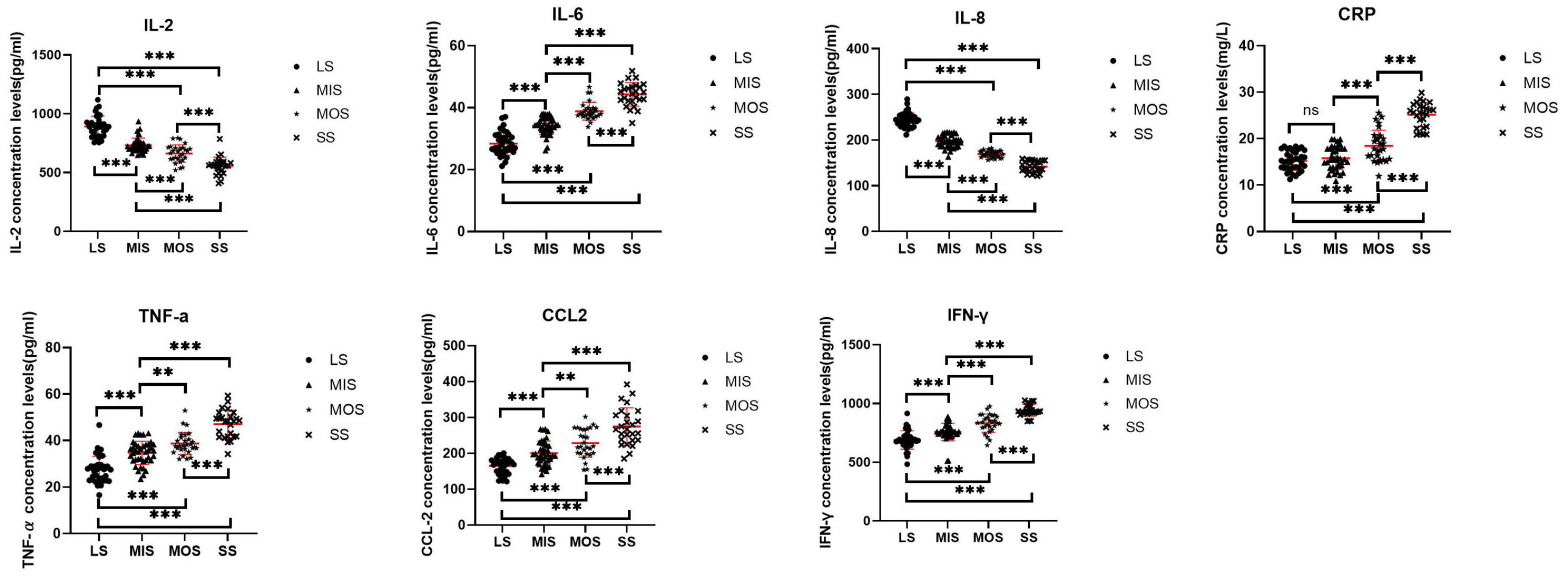
The concentration of serum inflammatory cytokines were compared between the four groups. The red line represents the M±SD. LS - Low suicide risk; MIS - Mild suicide risk; MOS - Moderate suicide risk; SS - Severe suicide risk; ns – not significant (P>0.05). *- significant correlation (P<0.05). **- significant correlation (P<0.01). ***- significant correlation (P<0.001).

The results of one-way ANOVA showed that the levels of IL-2, IL-6, IL-8, CRP, TNF-α, CCL2, and IFN-γ were significantly different among the four groups (all p<0.001) (see **Figure 2**). *Post-hoc* pairwise comparisons showed that the SS group of IL - 2 and IL - 8 were significantly lower than the MOS group (all p<0.001), the MOS group significantly below the MIS group (all p<0.001), the MIS group of these significantly lower than that of LS group (all p<0.001). In the IL-6, TNF-α, CCL2, and IFN-γ levels, the SS group was significantly higher than the MOS group (all p<0.001), the MOS group was significantly higher than the MIS group (p<0.001, p=0.003, p=0.003, p<0.001), and the MIS group was significantly higher than the LS group (all p<0.001). The CRP levels of the SS group were significantly higher than that of the MOS group (p<0.001), and the MOS group was significantly higher than that of the MIS group (p<0.001), but there was no significant difference between MIS and LS group (p=0.337).

### 3.4 Correlation analysis between levels of inflammatory cytokines and suicide risk in the MDD group

The correlation analysis results are shown in (**Table 2**). There was a significant negative correlation between age and IFN-γ(p<0.05, R^2^=(0.033), but it was not biologically significant because R^2^<0.47. There was no significant correlation between age and other factors (all p>0.05, R^2^<0.47).There was no significant relation between the years of education and other factors (all p>0.05, R^2^<0.47). Suicide risk was negatively correlated with IL-2 and IL-8 (all P<0.01, R^2 =^ 0.726, 0.920), and positively correlated with IL-6, CRP, TNF-α, CCL2, and IFN-γ (all P<0.01, R^2 =^ 0.794, 0.638, 0.659, 0.582, 0.632). IL-2 was positively correlated with IL-8 (P<0.01, R^2 =^ 0.699), and negatively correlated with IL-6, CRP, TNF-α, CCL2 and IFN-γ(all P<0.01, R^2 =^ 0.610, 0.468, 0.417, 0.343, 0.500). However, because R^2^ of CRP, TNF-α, CCL2 and IFN-γ was less than 0.47, it had no biological significance. IL-6 was significantly negatively correlated with IL-8(P<0.01,R^2 =^ 0.743), and positively correlated with CRP,TNF-α,CCL2 and IFN-γ(all P<0.01,R^2 =^ 0.545,0.613,0.402,0.461), but because R^2^ of CCL2 and IFN-γ was <0.47, it had no biological significance. IL-8 was negatively related to CRP, TNF-α, CCL2, and IFN-γ (all P<0.01, R^2 =^ 0.507,0.599,0.540,0.557). CRP was positively related to TNF-α, CCL2, and IFN-γ (all P<0.01, R^2 =^ 0.452, 0.449, 0.349). However, all R^2^<0.47 were not biologically significant. TNF-a was positively and significantly correlated with CCL2 and IFN-γ(all P<0.01, R^2 =^ 0.438, 0.403), but had no biological significance as all R^2^<0.47. CCL2 was positively and significantly correlated with IFN-γ(P<0.01, R^2 =^ 0.316), but due to R^2^<0.47. So there is no biological significance.

**Table 2:** Analysis of related variables(R(R2)P).

R(R^2^)^P^	Age	Years of Education	NGASR	IL-2	IL-6	IL-8	CRP	TNF-a	CCL2	IFN-γ
Age	1									
Years of Education	-0.029(0.001)	1								
NGASR	-0.140(0.020)	-0.053(0.003)	1							
IL-2	0.135(0.18)	0.098(0.010)	-0.852(0.726)^**^	1						
IL-6	-0.105(0.11)	-0.070(0.005)	0.891(0.794)^**^	-0.781(0.610)^**^	1					
IL-8	0.124(0.015)	0.030(0.001)	-0.959(0.920)^**^	0.836(0.699)^**^	-0.862(0.743)^**^	1				
CRP	-0.092(0.008)	-0.049(0.002)	0.799(0.638)^**^	-0.684(0.468)^**^	0.738(0.545)^**^	-0.712(0.507)^**^	1			
TNF-a	-0.069(0.005)	-0.129(0.017)	0.812(0.659)^**^	-0.646(0.417)^**^	0.783(0.613)^**^	-0.774(0.599)^**^	0.672(0.452)^**^	1		
CCL2	-0.164(0.027)	-0.145(0.021)	0.763(0.582)^**^	-0.586(0.343)^**^	0.634(0.402)^**^	-0.735(0.540)^**^	0.670(0.449)^**^	0.662(0.438)^**^	1	
IFN-γ	-0.181(0.033)^*^	-0.014(0.001)	0.795(0.632)^**^	-0.707(0.500)^**^	0.679(0.461)^**^	-0.746(0.557)^**^	0.591(0.349)^**^	0.635(0.403)^**^	0.562(0.316)^**^	1

* illustrate significant correlation (P<0.05).

** illustrate significant correlation (P<0.01).

### 3.5 Predictive relationship between inflammatory cytokines levels and suicide risk

The results of multiple linear regression analysis are shown in (**Table 3**). The fitting degree of this model was good, R²=0.964>0.2, indicating that the calculation results were reliable. VIF values were all less than 10, so multicollinearity was not considered among the seven variables. After controlling for age, IL-2 and IL-8 significantly negatively predicted suicide risk (p<0.001,p=0.003). IL-6, CRP, TNF-α,CCL2, and IFN-γ all significantly positively predicted suicide risk (p=0.026,p<0.001,p<0.001,p=0.030,p=0.010).Regression equation: Suicide risk=8.805–0.002*IL-2 + 0.061*IL-6–0.044*IL-8 + 0.106*CRP+0.026*TNF-a+0.005*CCL2 + 0.004*IFN-γ.

**Table 3:** Predictive relationship between variables.

	B	Sb	Beta	t	p	VIF	R²	F
IL-2	-0.002	0.001	-0.074	-2.249	<0.001	3.865	0.964	495.552
IL-6	0.061	0.020	0.116	3.082	0.026	5.128		
IL-8	-0.044	0.004	-0.502	-11.102	0.003	7.354		
CRP	0.106	0.021	0.140	5.078	<0.001	2.743		
TNF-a	0.026	0.012	0.065	2.194	<0.001	3.126		
CCL2	0.005	0.002	0.070	2.625	0.030	2.565		
IFN-γ	0.004	0.001	0.125	4.802	0.010	2.433		

MDD-major depressive disorder; HC-healthy controls.

## 4 Discussion

Firstly, in this study, we measured mental health status and concentrations of 10 peripheral blood inflammatory cytokines in 130 MDD patients and 130 healthy controls. Regarding mental health status, we found that the risk of suicide and the severity of depressive symptoms were significantly higher in MDD patients than in healthy people, which is consistent with previous studies (29). Depression has always been considered as an important cause of suicide, and anxiety symptoms often appear in patients with depression, and there is a positive correlation between them. More severe depressive symptoms are often accompanied by more severe anxiety symptoms (30). In terms of inflammatory cytokines, we found elevated serum levels of IL-6, TNF-α (31), CRP, and CCL2 in MDD patients, and we found no difference in IL-1β levels, which is generally consistent with previous studies (7, 9, 32). In addition, we found increased IFN-γ levels and decreased IL-2 and IL-8 levels in MDD patients. Our study found no difference in IL-10 and CXCL-1 levels. This is in contrast to previous studies. A meta-analysis found that patients with MDD had lower IFN-γ levels than healthy people, while IL-2 and IL-8 levels were not significantly changed (32). The reasons for the inconsistent results may be due to differences in study subjects, sample size, measurement tools, and geographical differences. Studies have found that peripheral blood inflammatory cytokines in patients with major depression can enter the brain and interact with all the pathophysiological causes of depression, such as neurotransmitter metabolism, neuroendocrine function, and neuroplasticity. In addition, the activation of inflammation in the brain can lead to the reduction of neurotrophic support in the brain, the change of glutamate release or reuptake, and the effect of oxidative stress, resulting in excitotoxicity and loss of glial cell components. It is worth noting that IL-6 and IL-1β have been shown to affect the pathophysiology of MDD by over-releasing corticotropin-releasing hormone and promoting glucocorticoid receptor resistance, which is likely to impair the negative feedback regulation of the HPA axis (33), and IL-6 may also activate the classical and trans-signaling pathways. Both pathways have combined anti-inflammatory and pro-inflammatory effects, respectively (34).

Secondly, the comparison of inflammatory cytokines in MDD patients grouped by suicide risk showed that with the increase of suicide risk, the concentration levels of IL-6, TNF-α, CCL2, and IFN-γ in peripheral blood gradually increased, while the concentration levels of IL-2 and IL-8 gradually decreased. In addition, for CRP, the SS group was higher than the MOS group, which in turn was higher than the MIS group. It is suggested that CRP may play an important role in the risk of suicide only in moderate to severe cases, but not in mild to moderate cases. However, we found no difference between the MIS group and the LS group in CRP. Most of our results are consistent with previous studies (9, 14, 35–37). The differences are mainly as follows: on the one hand, some studies found that IL-2 increases with its receptor in suicidal patients (11). On the other hand, several studies found no difference in IL-6 (33, 38), il-8 (39), TNF-α (40, 41), and CRP (9) between suicidal patients and healthy people. The reason for the inconsistent results may be that most of their research objects focused on MDD patients with or without suicide risk, while we studied MDD patients with different severity of suicide risk, and the differences in sample size and blood drawing methods may also cause differences.

Finally, it was found that the decrease of IL-2 and IL-8 and the increase of IL-6, CRP, TNF-α, CCL2, and IFN-γ all predicted the increase in suicide risk. This is in line with the findings of İlknur Ucuz (42). This study also demonstrated that specific inflammatory cytokines in plasma are predictors of suicide risk.

Therefore, the clinical importance of our results is that we can measure the concentration levels of specific peripheral blood inflammatory cytokines in MDD patients to predict patients who may be at high risk of suicide and to carry out further examination and intervention to reduce the suicide rate of MDD patients.

## 5 Limitations

This study investigated the internal mechanism of inflammatory cytokines in MDD patients with different degrees of suicide risk. Although there are significant advantages, there are some limitations: (1) This study used a self-evaluation scale, so the subjective differences of patients cannot be excluded, and each person’s understanding of the scale is different, which will cause certain errors. In addition, there are few studies on the reliability and validity of the Chinese version of NGASR. (2) It may cause some error to determine whether the patient did not take the medication based on the patient’s medical records and the oral statements of the patient and his family. (3)Each patient with MDD has a different onset of symptoms, and there may be an association between symptom duration and inflammatory cytokines, which could introduce some error. (4)The sample size is small and the experimental results may not be fully representative of the target group. (5) Blood was not drawn from every participant at the same time of day, because some inflammatory cytokines have circadian rhythms, and drawing blood at different times can be errant. (6) Lack of follow-up studies.

## 6 Conclusion

The purpose of this study was to investigate the relationship between suicide risk and specific inflammatory markers in MDD patients and provide references for the literature. We mainly drew the following conclusions: (1) MDD patients had higher levels of IL-6, CRP, TNF-α, CCL2, IFN-γ and lower levels of IL-2, IL-8. (2) IL-2, IL-6, IL-8, CRP, TNF-α, CCL2, and IFN-γ were all associated with suicide risk in MDD patients. (3) The higher the levels of IL-6, CRP, TNF-α,CCL2, and IFN-γ, and the lower the levels of IL-2 and IL-8 in MDD patients, the higher the suicide risk. This study highlights the importance of paying attention to the concentration of inflammatory cytokines in MDD patients to predict their risk of suicide. Our findings enrich our understanding of the inflammatory aspects of suicide risk in MDD patients and fill a gap in the research on the internal mechanism of suicide risk in MDD patients. However, more similar studies are needed in the future to confirm this idea and further clarify the specific pathways behind the role of inflammatory cytokines in suicide risk. Beyond that, future work needs to examine whether reduced inflammation leads to a reduced risk of suicide.

## Data availability statement

The raw data supporting the conclusions of this article will be made available by the authors, without undue reservation.

## Ethics statement

The studies involving humans were approved by The ethics committee of the First Affiliated Hospital of Chongqing Medical University. The studies were conducted in accordance with the local legislation and institutional requirements. The participants provided their written informed consent to participate in this study.

## Author contributions

YY: Conceptualization, Data curation, Formal analysis, Investigation, Methodology, Software, Writing – original draft, Writing – review & editing. KG: Formal analysis, Investigation, Methodology, Resources, Validation, Writing – original draft. JL: Conceptualization, Funding acquisition, Project administration, Resources, Supervision, Validation, Visualization, Writing – review & editing.

## Supplementary material

Supplementary Table 1 Comparison of inflammatory cytokines levels test results between the two groups. MDD-major depressive disorder; HC-healthy controls.

Supplementary Table 2 Inflammatory cytokines levels in depression patients grouped by suicide risk. LS - Low suicide risk; MIS - Mild suicide risk; MOS - Moderate suicide risk; SS - Severe suicide risk.
