# Supplementary material for: Relationship between serum inflammatory cytokines and suicide risk in patients with major depressive disorder
Source: Front Psychiatry. 2024 Jun 27;15:1422511. doi: 10.3389/fpsyt.2024.1422511 (PMC11236750; doi:10.3389/fpsyt.2024.1422511)
Supplement: Supplementary Table 1 — Comparison of inflammatory cytokines levels test results between the two groups. MDD-major depressive disorder; HC-healthy controls. [file Table_1.docx]

Supplementary **Table 1**．****Comparison of inflammatory** cytokines **levels test results between the two groups****

| **inflammatory cytokines** | **M±SD** | **M±SD** | ***p-*value** |
| --- | --- | --- | --- |
| IL-1β | 51.98±11.87 | 50.49±11.61 | 0.305 |
| IL-2 | 725.96±140.87 | 829.74±106.58 | <0.001 |
| IL-6 | 35.58±6.57 | 33.95±5.19 | 0.027 |
| IL-8 | 193.00±39.39 | 211.60±35.89 | <0.001 |
| IL-10 | 525.42±126.05 | 520.37±120.34 | 0.741 |
| CRP | 18.07±4.54 | 15.28±2.40 | <0.001 |
| TNF-a | 36.09±8.67 | 33.18±10.12 | 0.013 |
| CXCL-1 | 4.83±0.97 | 4.75±0.97 | 0.507 |
| CCL2 | 211.57±114.65 | 199.12±40.56 | 0.035 |
| IFN-γ | 791.31±120.34 | 726.04±119.87 | <0.001 |

**Abbreviations:** MDD-**major depressive disorder**；HC-healthy controls.
