# Supplementary material for: Relationship between serum inflammatory cytokines and suicide risk in patients with major depressive disorder
Source: Front Psychiatry. 2024 Jun 27;15:1422511. doi: 10.3389/fpsyt.2024.1422511 (PMC11236750; doi:10.3389/fpsyt.2024.1422511)
Supplement: Supplementary Table 2 — Inflammatory cytokines levels in depression patients grouped by suicide risk. LS - Low suicide risk; MIS - Mild suicide risk; MOS - Moderate suicide risk; SS - Severe suicide risk. [file Table_2.docx]

Supplementary **Table 2**．Inflammatory cytokines levels in depression patients grouped by suicide risk

|  | **LS(n=36)** | **MIS (n=40)** | **MOS (n=28)** | **SS (n=26)** | **Omnibus Test** | | | ****Post-hoc** statistics** | | |
| --- | --- | --- | --- | --- | --- | --- | --- | --- | --- | --- |
| **Cytokines** | **M±SD** | **M±SD** | **M±SD** | **M±SD** | ***F*** | ***df*** | ***P*** | **(I)Groups** | **(J)Groups** | ***p*** |
| IL-2 | 891.87±87.57 | 731.86±61.35 | 662.09±73.93 | 555.96±74.55 | 111.21 | 3 | <0.001 | LS | MIS | <0.001 |
|  |  |  |  |  |  |  |  |  | MOS | <0.001 |
|  |  |  |  |  |  |  |  |  | SS | <0.001 |
|  |  |  |  |  |  |  |  | MIS | MOS | <0.001 |
|  |  |  |  |  |  |  |  |  | SS | <0.001 |
|  |  |  |  |  |  |  |  | MOS | SS | <0.001 |
| IL-6 | 28.38±3.70 | 34.13±2.77 | 38.84±2.87 | 44.27±3.79 | 130.09 | 3 | <0.001 | LS | MIS | <0.001 |
|  |  |  |  |  |  |  |  |  | MOS | <0.001 |
|  |  |  |  |  |  |  |  |  | SS | <0.001 |
|  |  |  |  |  |  |  |  | MIS | MOS | <0.001 |
|  |  |  |  |  |  |  |  |  | SS | <0.001 |
|  |  |  |  |  |  |  |  | MOS | SS | <0.001 |
| IL-8 | 244.18±15.96 | 197.03±13.05 | 169.08±7.13 | 141.69±7.13 | 363.91 | 3 | <0.001 | LS | MIS | <0.001 |
|  |  |  |  |  |  |  |  |  | MOS | <0.001 |
|  |  |  |  |  |  |  |  |  | SS | <0.001 |
|  |  |  |  |  |  |  |  | MIS | MOS | <0.001 |
|  |  |  |  |  |  |  |  |  | SS | <0.001 |
|  |  |  |  |  |  |  |  | MOS | SS | <0.001 |
| CRP | 15.22±2.05 | 15.79±2.42 | 18.42±3.40 | 25.15±2.60 | 88.39 | 3 | <0.001 | LS | MIS | 0.337 |
|  |  |  |  |  |  |  |  |  | MOS | <0.001 |
|  |  |  |  |  |  |  |  |  | SS | <0.001 |
|  |  |  |  |  |  |  |  | MIS | MOS | <0.001 |
|  |  |  |  |  |  |  |  |  | SS | <0.001 |
|  |  |  |  |  |  |  |  | MOS | SS | <0.001 |
| TNF-a | 27.56±5.70 | 34.73±5.06 | 38.76±4.76 | 47.13±5.97 | 69.90 | 3 | <0.001 | LS | MIS | <0.001 |
|  |  |  |  |  |  |  |  |  | MOS | <0.001 |
|  |  |  |  |  |  |  |  |  | SS | <0.001 |
|  |  |  |  |  |  |  |  | MIS | MOS | 0.003 |
|  |  |  |  |  |  |  |  |  | SS | <0.001 |
|  |  |  |  |  |  |  |  | MOS | SS | <0.001 |
| CCL2 | 164.90±22.71 | 200.62±33.51 | 228.38±39.56 | 274.94±51.74 | 48.03 | 3 | <0.001 | LS | MIS | <0.001 |
|  |  |  |  |  |  |  |  |  | MOS | <0.001 |
|  |  |  |  |  |  |  |  |  | SS | <0.001 |
|  |  |  |  |  |  |  |  | MIS | MOS | 0.003 |
|  |  |  |  |  |  |  |  |  | SS | <0.001 |
|  |  |  |  |  |  |  |  | MOS | SS | <0.001 |
| IFN-γ | 689.54±78.74 | 757.78±74.28 | 831.87±77.38 | 940.13±49.88 | 66.56 | 3 | <0.001 | LS | MIS | <0.001 |
|  |  |  |  |  |  |  |  |  | MOS | <0.001 |
|  |  |  |  |  |  |  |  |  | SS | <0.001 |
|  |  |  |  |  |  |  |  | MIS | MOS | <0.001 |
|  |  |  |  |  |  |  |  |  | SS | <0.001 |
|  |  |  |  |  |  |  |  | MOS | SS | <0.001 |

**Abbreviations: LS - Low suicide risk; MIS - Mild suicide risk;MOS - Moderate suicide risk; SS - Severe suicide risk.**
